# Development and implementation of a commissioned pathway for the identification and stratification of liver disease in the community

**DOI:** 10.1136/flgastro-2019-101177

**Published:** 2019-06-26

**Authors:** Jane Chalmers, Emilie Wilkes, Rebecca Harris, Lucy Kent, Sonali Kinra, Guru Aithal, Mary Holmes, Jeanette Johnson, Joanne Morling, Indra Neil Guha

**Affiliations:** 1 NIHR Nottingham Biomedical Research Centre, Nottingham University Hospitals NHS Trust and the University of Nottingham, Nottingham, UK; 2 Nottingham Digestive Diseases Centre, Nottingham, UK; 3 Northern Lincolnshire and Goole Hospitals NHS Foundation Trust, Grimsby, Lincolnshire, UK; 4 Greater Nottingham Clinical Commissioning Group, Nottingham, UK; 5 Division of Epidemiology and Public Health, University of Nottingham, Nottingham, UK

**Keywords:** liver function test, fatty liver, alcoholic liver disease

## Abstract

**Objective:**

To describe the development of the Nottingham liver disease stratification pathway, present a 12-month evaluation of uptake and stratification results, and compare the pathway with current British Society of Gastroenterology (BSG) guidelines.

**Design:**

A referral pathway between primary and secondary care for the detection and risk stratification of liver disease.

**Setting:**

Four Nottinghamshire clinical commissioning groups (700 000 population).

**Patients:**

Patients are referred to the pathway with (1) raised aspartate aminotransferase to alanine aminotransferase (AST:ALT) ratio, (2) harmful alcohol use or (3) risk or presence of non-alcoholic fatty liver disease (NAFLD).

**Interventions:**

We report on clinic attendance within secondary care for transient elastography (TE) and brief lifestyle intervention. The TE result is reported back to the general practitioner with advice on interpretation and referral guidance.

**Main outcome measures:**

Pathway uptake, patient characteristics, liver disease stratification results and stakeholder feedback.

**Results:**

Over the first 12 months 968 patients attended a TE clinic appointment, with raised AST:ALT ratio being the most common single reason for referral (36.9%). Of the total, 222 (22.9%) patients had an elevated liver stiffness (≥8 kPa), in whom 57 (25.7%) had a liver stiffness which was indicative of advanced chronic liver disease. If a traditional approach based on raised liver enzymes (BSG guidance) had been followed, 38.7% of those with significant liver disease (≥8 kPa) would have gone undetected among those referred for either NAFLD or raised AST:ALT ratio.

**Conclusions:**

Targeting patients with risk factors for chronic liver disease and stratifying them using TE can detect significant chronic liver disease above and beyond the approach based on liver enzyme elevation.

**Significance of this study**

**What is already known on this topic**

- With rising levels of obesity, diabetes and increasing alcohol intake, the number of those at risk of liver disease in the community is growing.
- Reliance on raised liver enzymes for detection and stratification of patients may miss significant liver disease.
- Stratification of patients at risk of liver disease is cost-effective to the National Health Service.

**What this study adds**

- The creation of a clinically commissioned, integrated referral pathway between primary and secondary care is both feasible and effective.
- Through a combination of traditional blood-based biomarkers, a risk factor approach and transient elastography, this pathway identifies disease above the current guidance.

**How might it impact on clinical practice in the foreseeable future**

- Different iterations of this integrated pathway designed to suit local need and risk factor burden may provide a more robust way to identify more disease early, enabling lifestyle modification and better prognosis for patients.

## Introduction

The need for early detection of liver disease in order to allow intervention and to change the course of the disease has been highlighted by three independent reports.^1–3^ With no nationally agreed assessment guidance, the approach used by general practitioners (GPs) to identify patients with chronic liver disease (CLD) varies widely. Current diagnostic pathways for the detection and onward referral of suspected CLD are based on raised liver enzymes, which lack accuracy and may result in delays to diagnosis.

Serum levels of alanine aminotransferase (ALT) have been used as an indicator of liver cell injury for about 50 years. It is however recognised that significant liver fibrosis exists in the context of normal ALT.^4–6^ Conversely, the prevalence of raised liver enzymes is high within general practice, and yield of liver disease diagnoses may be low.^7^ Latterly, several clinical scoring systems based on routine laboratory indices have been shown to identify advanced fibrosis in patients with liver disease. The aspartate aminotransferase to alanine aminotransferase (AST:ALT) ratio, BARD score and non-alcoholic fatty liver disease (NAFLD) fibrosis scores all have a high negative predictive value (>92%) for advanced fibrosis and as such can be used as tools to identify those most at risk of disease.^8^

Considering this, we developed a pathway to target the risk factors which underpin the two most common causes of liver disease in the UK, alcohol-related liver disease and NAFLD. The second conceptual change we made was to allow direct access to non-invasive tests of liver fibrosis within primary care. We chose transient elastography (TE), as this has been extensively validated against liver biopsy and gives an immediate test result permitting a prompt intervention with lifestyle advice.

Our first pilot study, performed in a suburban area of Nottingham, found 10% of patients had harmful alcohol or type 2 diabetes as risk factors for liver disease.^9^ Targeting these risk factors resulted in finding potentially significant liver disease (TE ≥8 kPa) in 26.8% and cirrhosis (TE ≥15 kPa) in 3% of those tested. This represented a 140% increase in diagnoses of cirrhosis within this population, and 72.8% of this cohort had normal liver enzymes. Thus, we showed feasibility of a risk factor-based approach to identifying and stratifying liver disease across primary and secondary care. Furthermore, a formal economic evaluation showed this approach is cost-effective.^10^

In 2016, through discussion with four clinical commissioning groups (CCGs) in Nottingham, we negotiated an agreed pathway for liver disease stratification of patients at risk of liver disease. This included patients with a history of harmful alcohol use, risk factors for NAFLD or an AST:ALT ratio >0.8 in the context of raised liver enzymes. This paper describes the Nottingham liver disease stratification pathway, a 12-month evaluation of uptake and stratification results, and compares the pathway with existing clinical guidance from the British Society of Gastroenterology (BSG).^11^

## Methods

### Referral process for the pathway

**Figure 1:**
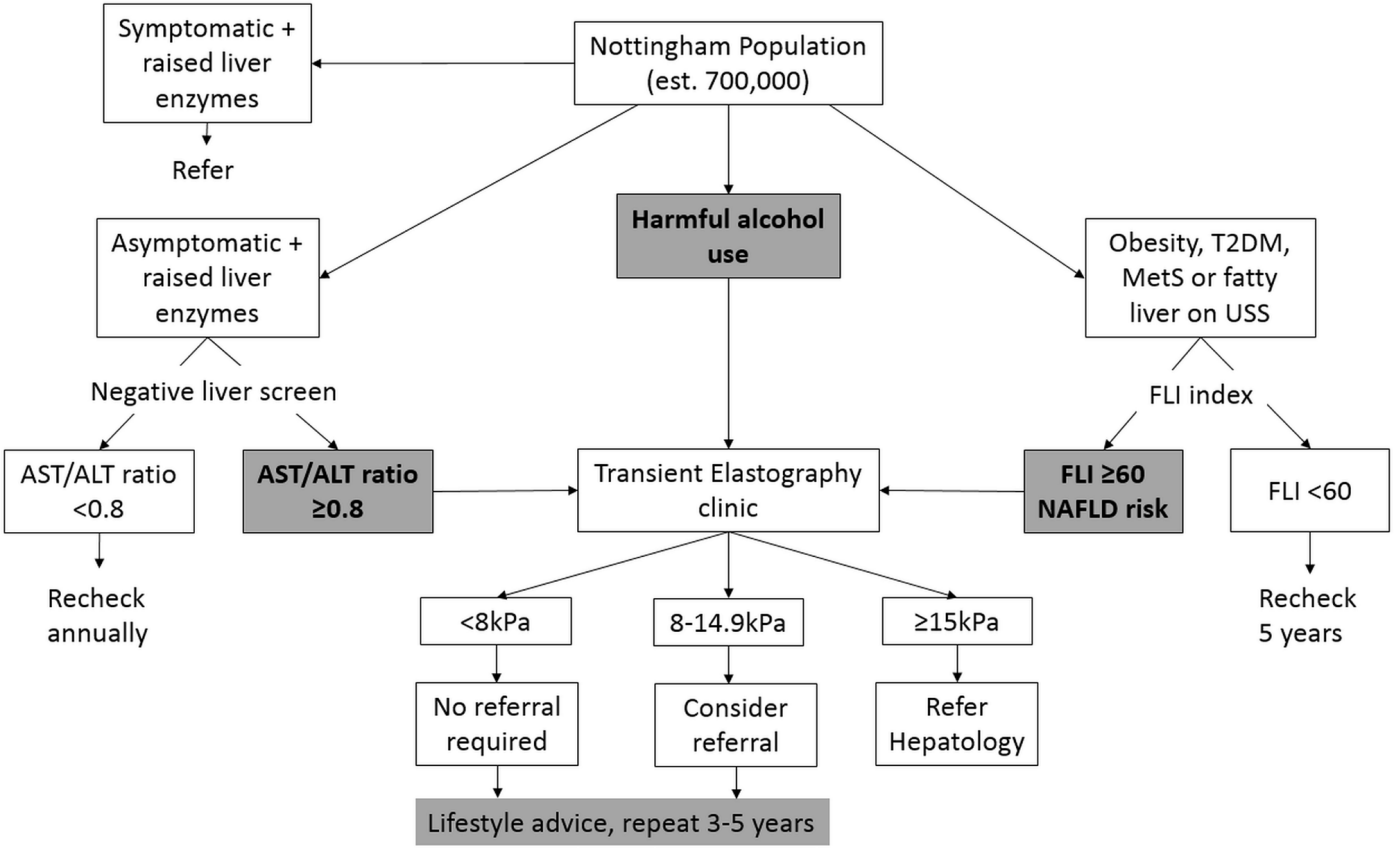
Nottingham liver disease stratification referral pathway. ALT, alanine aminotransferase; AST, aspartate aminotransferase; FLI, fatty liver index; MetS, metabolic syndrome; NAFLD, non-alcoholic fatty liver disease; T2DM, type 2 diabetes mellitus; USS, ultrasound.

Referral to the pathway (figure 1) is available to all patients attending their GP within the four CCG catchment areas under the following criteria:

- Harmful alcohol use (>50 units/week for men and >35 units/week for women, or presence of Read codes related to alcohol misuse).
- An AST:ALT ratio >0.8 in the context of raised liver enzymes.
- Risk of NAFLD (a fatty liver index (FLI) ≥60,^12^ and (1) presence of obesity, type 2 diabetes or metabolic syndrome, or (2) evidence of NAFLD on imaging).

GPs access the pathway by electronic referral through a local online system: ‘Integrated Clinical Environment’ or ‘ICE’. The referrals are then processed by the TE clinic team (details below) and an appointment is provided directly to the patient. The TE result is reported back to the GP with advice on interpretation and referral guidelines. Patients who have a TE result of <8 kPa are considered below referral threshold and are recommended to have a repeat TE in 5 years if still indicated. Patients with a TE result between 8 kPa and 14.9 kPa should be considered for referral to hepatology services (if not, repeat TE in 3 years if indicated). For patients with a TE result ≥15 kPa, referral to the local hepatology service is recommended.

### TE clinic

The TE clinic is nurse-led at the Queen’s Medical Centre, Nottingham University Hospitals NHS Trust. The clinic runs from 08:00 to 16:00, 4 days a week with ten 30 min patient appointments per day. The clinic is staffed by a band 6 nurse with support from a band 3 healthcare assistant (both trained to perform TE and deliver a brief intervention).

Patients who attend the clinic undergo basic anthropometric measurements of height (cm), weight (kg) and blood pressure (mm Hg). Smoking status and alcohol intake are documented along with the results of any basic blood and liver screen that have been done prior to attendance. Patients undergo TE using FibroScan (Echosens, Paris, France), with the result being a median of 10 readings and validity presented as IQR. They receive a brief intervention, regardless of the result. This intervention includes signposting to local alcohol and weight management services as appropriate.

### Comparison with the BSG guidelines

At present there are no robust guidelines regarding patient selection for assessment for significant liver disease. The National Institute for Health and Care Excellence (NICE) recommends the use of the enhanced liver fibrosis (ELF) test in patients with evidence of NAFLD, but does not outline any case finding strategies. Current BSG guidance recommends assessment for fibrosis using one of the blood-based parameters in the community (FIB4 or NAFLD Fibrosis Score) in patients with suspected NAFLD, but only in the context of raised liver enzymes.

In order to examine the potential additional benefit of our case finding approach using risk factors over a traditional approach, we have compared the stratification results of patients through the Nottingham pathway (excluding those referred with harmful alcohol intake) with the stratification results should the current BSG guidance have been followed, that is, assessing only those with raised liver enzymes.

### Patient and GP feedback

All patients who attend the TE clinic are requested to fill in an anonymous feedback form related to the service. This includes questions about the appointment, the staff and their understanding of the outcome of the appointment.

Feedback was also collected from GP practices which access the pathway. This was done through a postal questionnaire that comprised three questions:

- Do the Nottingham liver disease stratification pathway guidelines make sense?
- Is the Nottingham liver disease stratification pathway referral process easy to use?
- Has the Nottingham liver disease stratification pathway improved patient care?

For each question GPs were asked to rate their response from 1 (not at all) to 4 (totally). Two copies of the questionnaire were sent to each of the 110 GP practices involved.

### Analysis

Analyses will evaluate the data collected between 1 September 2016 and 30 August 2017.

We report on service uptake (clinic attendance rates and referral reason), patient characteristics (with/without elevated TE), liver disease stratification results (including comparison with existing guidelines), secondary care referral numbers and stakeholder feedback.

Descriptive data are presented for clinic attendance, referral patterns and waiting times, referral reason (defined as harmful alcohol use, AST:ALT ratio >0.8, NAFLD or in combination), and stratification of liver disease (defined as no significant liver disease TE <8 kPa, significant liver disease TE 8–14.9 kPa, advanced liver disease ≥15 kPa). Categorical data are presented as number (percentage). Continuous data are presented as mean (SD) for normally distributed data and median (range) for non-normal data.

Univariate analysis to compare the characteristics of participants with/without elevated TE was undertaken using Student’s t-test (normal continuous), Mann-Whitney U test (non-normal continuous) and χ^2^ test (categorical).

Statistical analysis was performed using STATA V.15.

## Results

### Service uptake

In total, 968 patients attended a TE clinic appointment through the Nottingham liver disease stratification pathway between September 2016 and August 2017. The number of patients who attended the clinic increased from 24 in month 1, to 129 in month 12 (online supplementary file). The average waiting time for a TE clinic appointment was 20 days, and the percentage of used appointments to which a patient did not attend ranged between 5% and 15% per month.

10.1136/flgastro-2019-101177.supp1 Supplementary data

### Patient characteristics

The baseline characteristics and TE results of these patients are outlined in table 1.

**Table 1:** Baseline characteristics and TE results of all patients referred through the Nottingham liver disease stratification pathway

Variable		Results N=968
Male gender	n (%)	470 (49)
Age (years)	Mean (SD)	56.3 (13.7)
Body mass index (kg/m^2^)	Mean (SD)	31.2 (6.9)
Current alcohol intake >14 units/week	n (%)	304 (31.4)
Current smoker	n (%)	276 (28.5)
Referral criteria		
Harmful alcohol use only	n (%)	94 (9.7)
AST:ALT ratio >0.8 only	n (%)	357 (36.9)
NAFLD risk only	n (%)	223 (23)
Combination	n (%)	267 (27.6)
No criteria met	n (%)	27 (2.8)
Bilirubin (μmol/L)	Mean (SD)	12 (8)
AST (U/L)	Median (IQR)	44 (28)
ALT (U/L)	Median (IQR)	49 (28)
Albumin (g/L)	Mean (SD)	42 (4)
Platelets (×10^9^/L)	Mean (SD)	265 (80)
FibroScan result		
<8.0 kPa	n (%)	740 (76.5)
8.0–14.9 kPa	n (%)	165 (17)
≥15 kPa	n (%)	57 (5.9)
Technical failure	n (%)	6 (0.6)

ALT, alanine aminotransferase; AST, aspartate aminotransferase; NAFLD, non-alcoholic fatty liver disease; TE, transient elastography.

**Figure 2:**
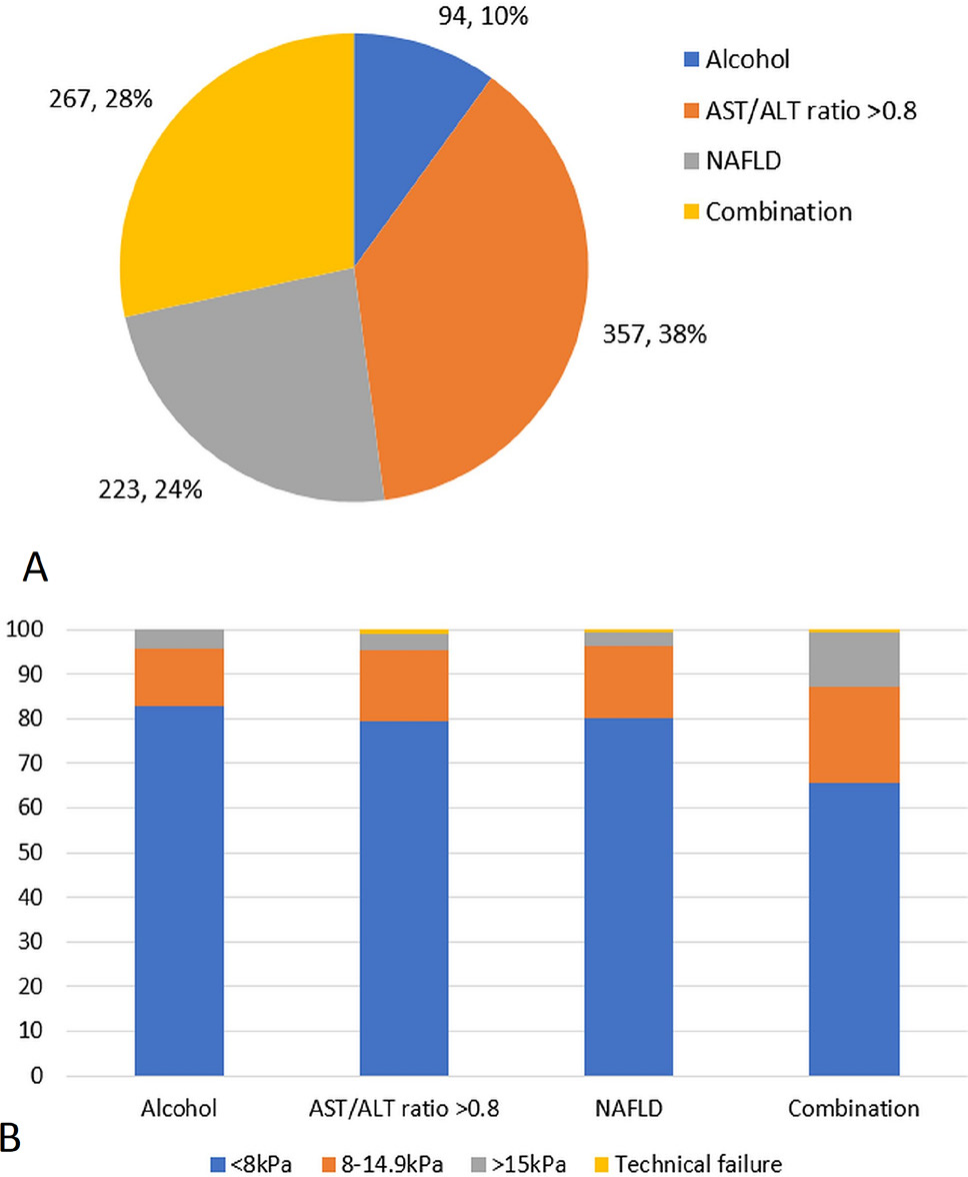
(A) Distribution of referral criteria to Nottingham liver disease stratification pathway (excluding those who did not meet the criteria). (B) Transient elastography results of these patients according to the referral criteria. ALT, alanine aminotransferase; AST, aspartate aminotransferase; NAFLD, non-alcoholic fatty liver disease.

Of the patients, 941 (97.2%) met one or more of the referral criteria. The most common reason for referral was AST:ALT ratio >0.8 (357, 36.9%; figure 2). A large proportion of patients were referred with either NAFLD (223, 23%) or a combination of risk factors (267, 27.6%). Two hundred and thirteen (79.8%) of those referred for a combination of risk factors were for AST:ALT ratio >0.8 combined with either harmful alcohol intake or NAFLD. Fewer patients were referred with harmful alcohol use as a risk factor alone (94, 9.7%). Patients who did not meet the referral criteria were more likely to have a normal TE than those who had multiple risk factors (92.6% vs 65.5%, p=0.004). The average IQR for M and XL probe results was 0.89 kPa (0.14%) and 1.11 kPa (0.14%), respectively.

### Stratification of liver disease

#### Analysis of TE results that suggest significant liver disease

Liver stiffness was elevated in 222 (22.9%) patients (8–14.9 kPa, n=165 (74.3%); ≥15 kPa, n=57 (25.7%)). Patients with raised liver stiffness were older (59.9±13.0 vs 55.3±13.7, p<0.001) and had a higher body mass index (33.6±7.4 vs 30.3±6.6, p<0.001) than those with a normal liver stiffness (n=740).

Subsequently, 63 patients (38.2%) who had a TE of 8–14.9 kPa and 45 (78.9%) patients who had a TE of ≥15 kPa were referred to hepatology services. Of those with a TE of 8–14 kPa who were seen in clinic, 4 (2.4%) patients received a diagnosis of advanced CLD (Baveno VI criteria),^13^ giving a total of 61 (27.4%) patients.

#### Analysis of patients with normal liver enzymes: a comparison of Nottingham liver disease stratification pathway versus the BSG guidelines

Of those referred through the pathway, 96.8% (n=937) of patients had a documented ALT result, of which 78.5% were raised (ALT >35, n=736). There were, however, a significant proportion of patients (21.5%, n=201) who had a normal ALT.

**Figure 3:**
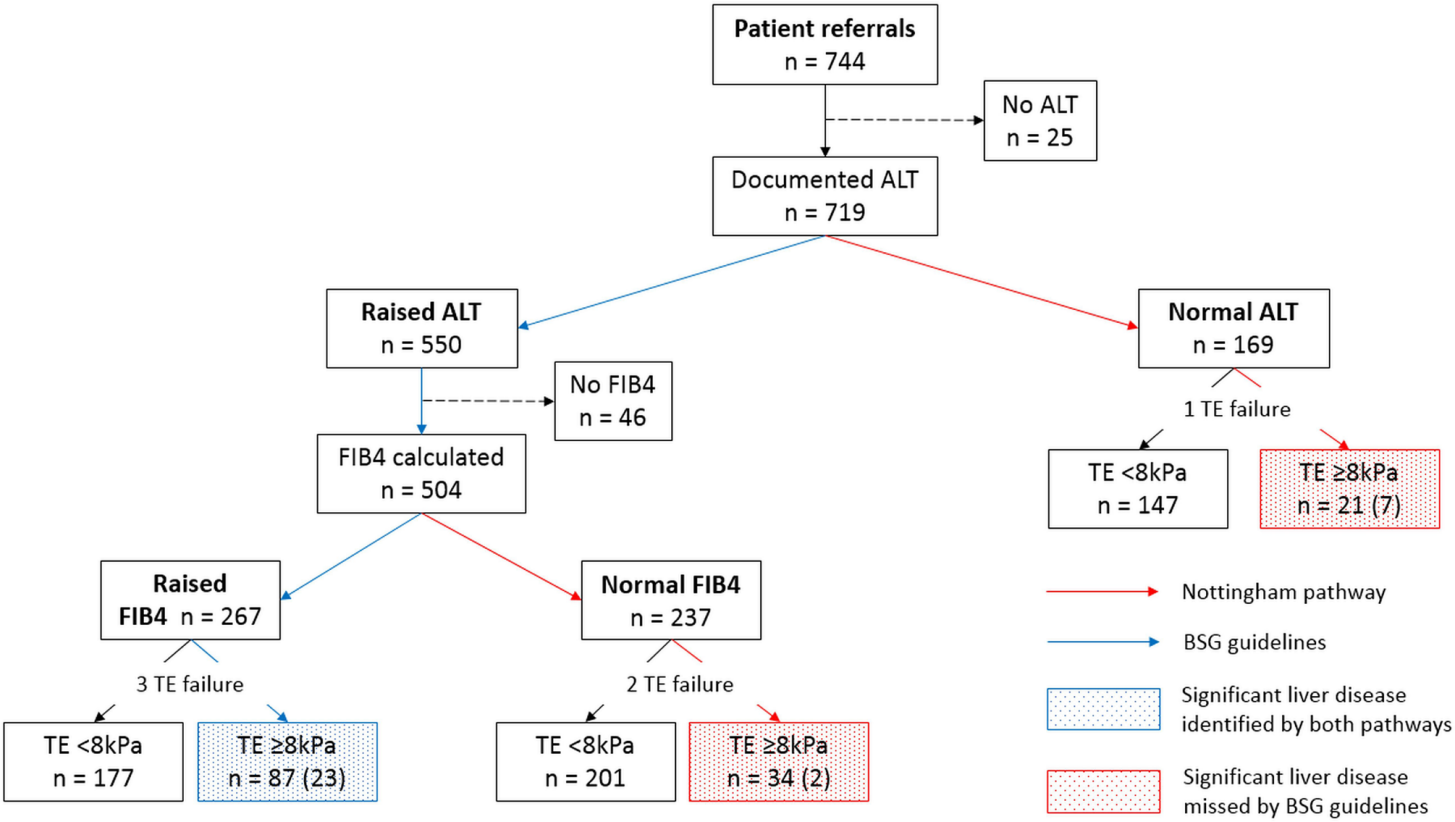
Stratification of patients through the Nottingham liver disease stratification pathway compared with standard stratification using the BSG guidelines (numbers in brackets ≥15 kPa). ALT, alanine aminotransferase; BSG, British Society of Gastroenterology; TE, transient elastography.

There were 744 patients referred to our pathway with either AST:ALT ratio >0.8 or NAFLD. Five hundred and fifty patients had a raised ALT, of whom FIB4 was available in 504. Through the Nottingham liver disease stratification pathway, 142 patients were found to have significant liver disease (TE ≥8 kPa), of whom 32 patients had advanced liver disease (TE ≥15 kPa). In contrast, only 87 patients with significant liver disease (23 patients with advanced liver disease) would have been identified if patients had been assessed only in the presence of abnormal liver enzymes. This signifies detection of an additional 55 patients using a risk factor approach, representing a relative increase in detection of 38.7% and an absolute increase in detection of 7.4% (55/744) of the total number of patients referred (figure 3).

### Stakeholder feedback

Out of 813 feedback questionnaires received between September 2016 and August 2017, 812 (99.9%) patients understood the reason for their appointment and 731 (89.9%) knew what would happen during the appointment. Of the patients 749 (92.1%) were either given a choice of appointment time or did not want/need a choice, and 781 (96%) found it easy to travel to the appointment. Overall, 804 (98.9%) of patients would recommend the service to family and friends.

Out of a possible 220 GP questionnaires, 41 (18.6%) were returned. The average score for whether the pathway guidelines made sense was 3.6 out of 4, whether the pathway was easy to use was 2.9 out of 4, and that it had improved patient care was 3.0 out of 4.

## Discussion

The Nottingham liver disease stratification pathway is to our knowledge the first commissioned pathway in routine clinical care to incorporate stratification of patients who are at risk of disease, even in the absence of raised liver enzymes or fatty liver on imaging, integrating primary and secondary care and enabling GP’s direct access to non-invasive assessment of liver fibrosis. This pathway was designed through a series of pilot studies and implemented through consultation with stakeholders and commissioners, and as such has resulted in a sustainable stratification model to suit local need which has the ability to diagnose disease above and beyond the current guidelines.

The prevalence of significant liver disease identified within primary care is variable depending on the diagnostic approach,^14^ but with rising levels of obesity, diabetes and increasing alcohol consumption the number of those at risk is growing. Current strategies for the identification of those with disease rely heavily on raised liver enzymes as a starting point.^11^ While the risk of disease is broadly higher in this group,^7^ studies show that 40%–74.6% of patients with a normal ALT have fibrosis on further assessment,^14^ meaning significant numbers of patients with disease may go undetected when stratified using a pathway that has raised liver enzymes as its first step. Newer models that automate the system of investigating patients with potential liver disease (which incorporate risk stratification tools) within primary care aim to improve access and efficiency of secondary care services,^15^ but still remain reliant on raised liver enzymes to qualify for assessment. The Nottingham liver disease stratification pathway starts by targeting risk factors and uses non-invasive markers of liver fibrosis to identify more disease while remaining cost-effective to the National Health Service (NHS).^10^ The importance of this is underlined by the fact that an additional 55 patients were found to have significant liver disease (TE ≥8 kPa) and would not have been diagnosed using established national guidelines.

A strength of the pathway is that its development through pilots to the final commissioning involved GPs, commissioners and other stakeholders. Conceptually targeting risk factors, making tools accessible to GPs and the incorporation of a brief intervention could be adapted to suit different populations. For example, the pathway has been tailored to evaluate patients within drug and alcohol misuse clinics, patients who are at risk of significant disease but may not present through primary care.

It is interesting to note that despite the intention to focus on risk factors and recognise the limitations of reliance on raised liver enzymes as a marker of CLD, 37% of patients are currently being referred with a raised AST:ALT ratio in the context of raised liver enzymes. Through continued effort to increase awareness, further evaluation of the newly established pathway and dissemination of our findings, efficiency of the pathway and the service can be improved further.

One limiting factor of this pathway is the inclusion of the FLI as a preliminary stratification tool in patients at risk of NAFLD. Our pilot data have shown that the prevalence of significant fibrosis in an unselected population with diabetes is not insignificant (31.5%).^16^ As such, a number of patients with disease may go unassessed. The FLI was included during the consultation phase of the pathway development—in line with the draft NICE guidelines of the time—and its use may be withdrawn as the pathway evolves. A further limitation of the evaluation of this pathway is that we are unable to assess its impact on GPs’ decision making. We do not have the data related to referral patterns from GPs and are unable to evaluate factors that influence patients’ choice and compliance, particularly in relation to low numbers of patients referred with harmful alcohol intake.

## Conclusion

The Nottingham liver disease stratification pathway is a locally commissioned pathway that has been shown to be effective by increasing the detection of significant liver disease when compared with an approach based on raised liver enzymes. The pathway demonstrates that providing GPs access to non-invasive markers to detect significant liver disease is feasible and has been received positively by both patients and physicians.
