# Supplementary figures and images for: Development and implementation of a commissioned pathway for the identification and stratification of liver disease in the community
Source: Frontline Gastroenterol. 2019 Jun 26;11(2):86–92. doi: 10.1136/flgastro-2019-101177 (PMC7025872; doi:10.1136/flgastro-2019-101177)

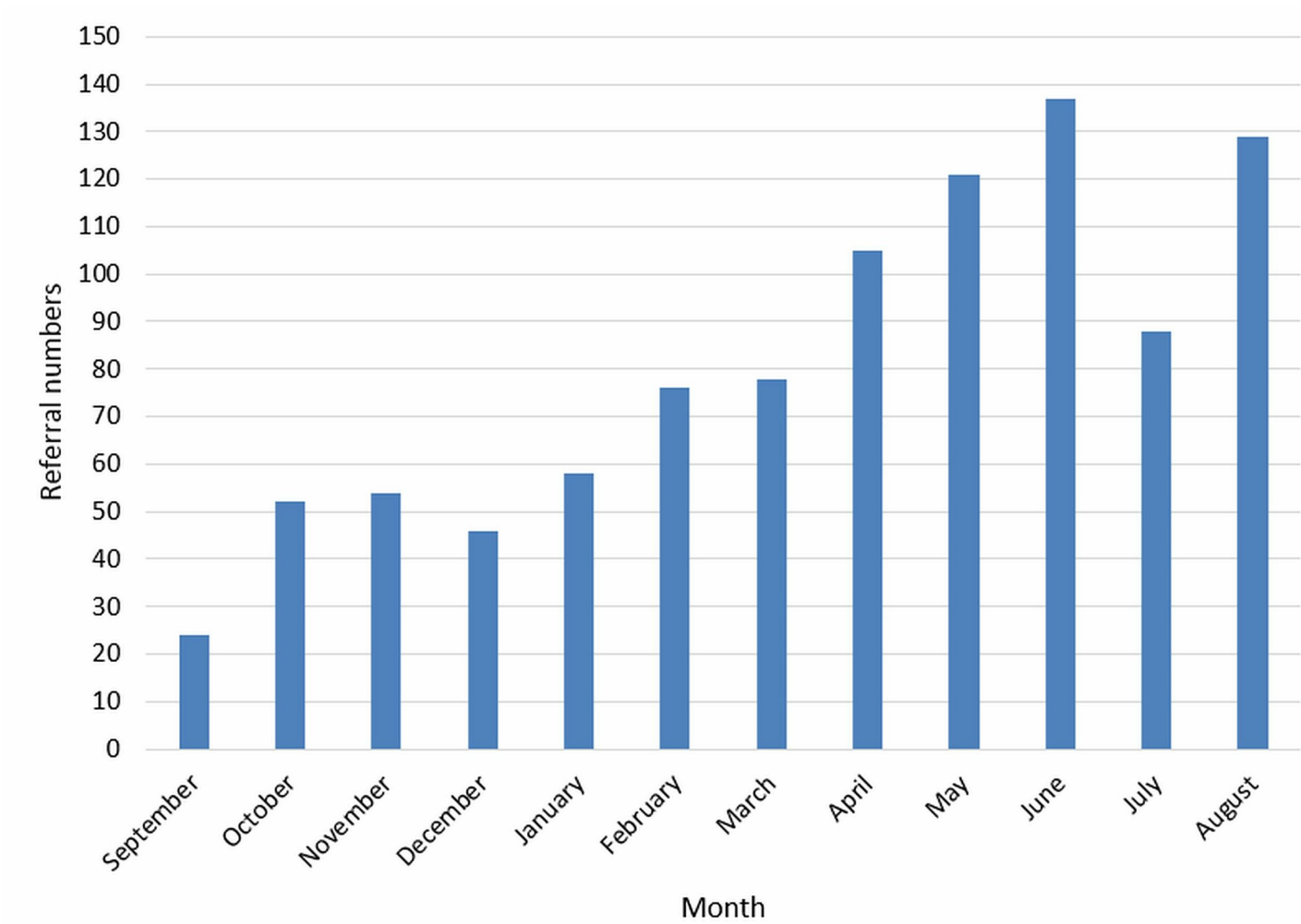

Supplement: Supplementary data [file flgastro-2019-101177supp001.pdf]
